# Supplementary material for: Modeling glioblastoma heterogeneity as a dynamic network of cell states
Source: Mol Syst Biol. 2021 Sep 16;17(9):e10105. doi: 10.15252/msb.202010105 (PMC8444284; doi:10.15252/msb.202010105)
Supplement: Supplementary file 5 — Source Data for Figure 3 [file MSB-17-e10105-s001.zip › Figure3A_sourcedata/GSEA_3065/hallmarks_state2.GseaPreranked.1623416269049/HALLMARK_P53_PATHWAY.html]

Details for gene set HALLMARK\_P53\_PATHWAY[GSEA]

|  || Dataset | state2 |
| Phenotype | NoPhenotypeAvailable |
| Upregulated in class | na\_neg |
| GeneSet | HALLMARK\_P53\_PATHWAY |
| Enrichment Score (ES) | -0.3901451 |
| Normalized Enrichment Score (NES) | -1.5631464 |
| Nominal p-value | 0.0 |
| FDR q-value | 0.015325687 |
| FWER p-Value | 0.107 |
Table: GSEA Results Summary

  

Fig 1: Enrichment plot: HALLMARK\_P53\_PATHWAY      
 Profile of the Running ES Score & Positions of GeneSet Members on the Rank Ordered List

  

| PROBE | GENE SYMBOL | GENE\_TITLE | RANK IN GENE LIST | RANK METRIC SCORE | RUNNING ES | CORE ENRICHMENT || 1 | PCNA |  |  | 16 | 0.595 | 0.0522 | No |
| 2 | TPRKB |  |  | 153 | 0.307 | 0.0655 | No |
| 3 | CCND2 |  |  | 227 | 0.263 | 0.0815 | No |
| 4 | RAD51C |  |  | 260 | 0.246 | 0.1004 | No |
| 5 | LDHB |  |  | 343 | 0.219 | 0.1114 | No |
| 6 | HINT1 |  |  | 418 | 0.196 | 0.1212 | No |
| 7 | TRAF4 |  |  | 708 | 0.149 | 0.1037 | No |
| 8 | EI24 |  |  | 828 | 0.135 | 0.1031 | No |
| 9 | RPS12 |  |  | 985 | 0.121 | 0.0974 | No |
| 10 | CCND3 |  |  | 1164 | 0.107 | 0.0879 | No |
| 11 | PDGFA |  |  | 1294 | 0.098 | 0.0830 | No |
| 12 | DNTTIP2 |  |  | 1370 | 0.094 | 0.0834 | No |
| 13 | RPL36 |  |  | 1430 | 0.091 | 0.0853 | No |
| 14 | DCXR |  |  | 1477 | 0.088 | 0.0883 | No |
| 15 | RPL18 |  |  | 1526 | 0.085 | 0.0909 | No |
| 16 | CCP110 |  |  | 1554 | 0.084 | 0.0956 | No |
| 17 | HRAS |  |  | 1748 | 0.075 | 0.0816 | No |
| 18 | GADD45A |  |  | 1791 | 0.073 | 0.0837 | No |
| 19 | JUN |  |  | 1805 | 0.072 | 0.0889 | No |
| 20 | TRIAP1 |  |  | 1827 | 0.071 | 0.0931 | No |
| 21 | NOL8 |  |  | 2020 | 0.064 | 0.0782 | No |
| 22 | APAF1 |  |  | 2127 | 0.060 | 0.0723 | No |
| 23 | NUDT15 |  |  | 2188 | 0.058 | 0.0710 | No |
| 24 | RACK1 |  |  | 2226 | 0.056 | 0.0722 | No |
| 25 | ZFP36L1 |  |  | 2320 | 0.053 | 0.0670 | No |
| 26 | PPM1D |  |  | 2605 | 0.044 | 0.0405 | No |
| 27 | S100A10 |  |  | 2608 | 0.044 | 0.0443 | No |
| 28 | WRAP73 |  |  | 2841 | 0.038 | 0.0228 | No |
| 29 | SP1 |  |  | 2960 | 0.035 | 0.0133 | No |
| 30 | ADA |  |  | 3009 | 0.034 | 0.0112 | No |
| 31 | PMM1 |  |  | 3320 | 0.027 | -0.0197 | No |
| 32 | TP53 |  |  | 3495 | 0.024 | -0.0362 | No |
| 33 | CDKN2AIP |  |  | 3686 | 0.021 | -0.0547 | No |
| 34 | PRKAB1 |  |  | 3878 | 0.018 | -0.0737 | No |
| 35 | CSRNP2 |  |  | 3977 | 0.016 | -0.0828 | No |
| 36 | RCHY1 |  |  | 3989 | 0.016 | -0.0825 | No |
| 37 | PITPNC1 |  |  | 4062 | 0.015 | -0.0890 | No |
| 38 | POM121 |  |  | 4110 | 0.014 | -0.0928 | No |
| 39 | POLH |  |  | 4128 | 0.014 | -0.0934 | No |
| 40 | IRAK1 |  |  | 4256 | 0.011 | -0.1060 | No |
| 41 | AEN |  |  | 4389 | 0.009 | -0.1194 | No |
| 42 | ELP1 |  |  | 4473 | 0.008 | -0.1276 | No |
| 43 | S100A4 |  |  | 4483 | 0.008 | -0.1279 | No |
| 44 | BAX |  |  | 4506 | 0.007 | -0.1296 | No |
| 45 | RXRA |  |  | 4592 | 0.006 | -0.1382 | No |
| 46 | FAM162A |  |  | 4622 | 0.005 | -0.1408 | No |
| 47 | MAPKAPK3 |  |  | 4691 | 0.004 | -0.1478 | No |
| 48 | CCNK |  |  | 4813 | 0.003 | -0.1605 | No |
| 49 | ISCU |  |  | 4841 | 0.002 | -0.1633 | No |
| 50 | CCNG1 |  |  | 4843 | 0.002 | -0.1632 | No |
| 51 | BAK1 |  |  | 4847 | 0.002 | -0.1633 | No |
| 52 | RAP2B |  |  | 4924 | 0.001 | -0.1714 | No |
| 53 | IER5 |  |  | 5041 | -0.001 | -0.1838 | No |
| 54 | HDAC3 |  |  | 5088 | -0.001 | -0.1887 | No |
| 55 | TAX1BP3 |  |  | 5161 | -0.003 | -0.1961 | No |
| 56 | XPC |  |  | 5262 | -0.004 | -0.2065 | No |
| 57 | VWA5A |  |  | 5352 | -0.006 | -0.2155 | No |
| 58 | PIDD1 |  |  | 5535 | -0.009 | -0.2343 | No |
| 59 | WWP1 |  |  | 5601 | -0.010 | -0.2404 | No |
| 60 | TRAFD1 |  |  | 5623 | -0.010 | -0.2417 | No |
| 61 | RALGDS |  |  | 5656 | -0.011 | -0.2441 | No |
| 62 | DRAM1 |  |  | 5714 | -0.012 | -0.2492 | No |
| 63 | KIF13B |  |  | 5735 | -0.012 | -0.2502 | No |
| 64 | CGRRF1 |  |  | 5966 | -0.016 | -0.2735 | No |
| 65 | RRP8 |  |  | 5983 | -0.016 | -0.2737 | No |
| 66 | RAB40C |  |  | 6018 | -0.017 | -0.2759 | No |
| 67 | SLC35D1 |  |  | 6040 | -0.017 | -0.2765 | No |
| 68 | HSPA4L |  |  | 6125 | -0.019 | -0.2839 | No |
| 69 | NINJ1 |  |  | 6212 | -0.020 | -0.2913 | No |
| 70 | UPP1 |  |  | 6249 | -0.021 | -0.2933 | No |
| 71 | CYFIP2 |  |  | 6362 | -0.023 | -0.3033 | No |
| 72 | PVT1 |  |  | 6385 | -0.023 | -0.3035 | No |
| 73 | DDIT3 |  |  | 6433 | -0.024 | -0.3064 | No |
| 74 | FBXW7 |  |  | 6536 | -0.026 | -0.3150 | No |
| 75 | EPHX1 |  |  | 6547 | -0.026 | -0.3137 | No |
| 76 | ABAT |  |  | 6574 | -0.027 | -0.3141 | No |
| 77 | PLK3 |  |  | 6607 | -0.027 | -0.3150 | No |
| 78 | ATF3 |  |  | 6737 | -0.030 | -0.3261 | No |
| 79 | PPP1R15A |  |  | 7004 | -0.037 | -0.3514 | No |
| 80 | SERTAD3 |  |  | 7057 | -0.038 | -0.3535 | No |
| 81 | RPS27L |  |  | 7139 | -0.040 | -0.3586 | No |
| 82 | BAIAP2 |  |  | 7169 | -0.041 | -0.3580 | No |
| 83 | FOS |  |  | 7222 | -0.043 | -0.3597 | No |
| 84 | MKNK2 |  |  | 7371 | -0.046 | -0.3714 | No |
| 85 | DDB2 |  |  | 7419 | -0.048 | -0.3721 | No |
| 86 | SESN1 |  |  | 7421 | -0.048 | -0.3679 | No |
| 87 | RGS16 |  |  | 7474 | -0.050 | -0.3690 | No |
| 88 | NDRG1 |  |  | 7538 | -0.051 | -0.3711 | No |
| 89 | ABCC5 |  |  | 7542 | -0.052 | -0.3667 | No |
| 90 | STOM |  |  | 7570 | -0.052 | -0.3649 | No |
| 91 | HEXIM1 |  |  | 7573 | -0.052 | -0.3603 | No |
| 92 | PHLDA3 |  |  | 7590 | -0.053 | -0.3572 | No |
| 93 | RAD9A |  |  | 7647 | -0.055 | -0.3583 | No |
| 94 | BLCAP |  |  | 7723 | -0.057 | -0.3612 | No |
| 95 | FDXR |  |  | 7726 | -0.057 | -0.3562 | No |
| 96 | SDC1 |  |  | 7783 | -0.059 | -0.3569 | No |
| 97 | TAP1 |  |  | 7963 | -0.066 | -0.3702 | No |
| 98 | TM4SF1 |  |  | 8150 | -0.074 | -0.3834 | Yes |
| 99 | CD82 |  |  | 8151 | -0.074 | -0.3766 | Yes |
| 100 | BTG1 |  |  | 8156 | -0.075 | -0.3703 | Yes |
| 101 | EPHA2 |  |  | 8159 | -0.075 | -0.3637 | Yes |
| 102 | TOB1 |  |  | 8169 | -0.076 | -0.3578 | Yes |
| 103 | ITGB4 |  |  | 8365 | -0.086 | -0.3709 | Yes |
| 104 | FUCA1 |  |  | 8399 | -0.089 | -0.3664 | Yes |
| 105 | ANKRA2 |  |  | 8486 | -0.095 | -0.3671 | Yes |
| 106 | TM7SF3 |  |  | 8495 | -0.095 | -0.3593 | Yes |
| 107 | TGFB1 |  |  | 8497 | -0.095 | -0.3508 | Yes |
| 108 | DDIT4 |  |  | 8601 | -0.103 | -0.3525 | Yes |
| 109 | PRMT2 |  |  | 8613 | -0.104 | -0.3442 | Yes |
| 110 | FOXO3 |  |  | 8616 | -0.104 | -0.3350 | Yes |
| 111 | MDM2 |  |  | 8707 | -0.112 | -0.3345 | Yes |
| 112 | TSC22D1 |  |  | 8743 | -0.115 | -0.3278 | Yes |
| 113 | NOTCH1 |  |  | 8763 | -0.118 | -0.3192 | Yes |
| 114 | CTSF |  |  | 8795 | -0.122 | -0.3115 | Yes |
| 115 | PLK2 |  |  | 8803 | -0.123 | -0.3011 | Yes |
| 116 | PLXNB2 |  |  | 8819 | -0.125 | -0.2914 | Yes |
| 117 | PERP |  |  | 8836 | -0.127 | -0.2816 | Yes |
| 118 | PTPRE |  |  | 8861 | -0.131 | -0.2722 | Yes |
| 119 | STEAP3 |  |  | 8934 | -0.139 | -0.2674 | Yes |
| 120 | TXNIP |  |  | 8987 | -0.147 | -0.2596 | Yes |
| 121 | PTPN14 |  |  | 9025 | -0.153 | -0.2498 | Yes |
| 122 | F2R |  |  | 9026 | -0.153 | -0.2359 | Yes |
| 123 | SLC7A11 |  |  | 9040 | -0.156 | -0.2231 | Yes |
| 124 | HMOX1 |  |  | 9084 | -0.163 | -0.2130 | Yes |
| 125 | IP6K2 |  |  | 9136 | -0.174 | -0.2027 | Yes |
| 126 | BTG2 |  |  | 9239 | -0.207 | -0.1949 | Yes |
| 127 | SEC61A1 |  |  | 9261 | -0.217 | -0.1774 | Yes |
| 128 | SLC3A2 |  |  | 9268 | -0.220 | -0.1581 | Yes |
| 129 | ZMAT3 |  |  | 9276 | -0.223 | -0.1387 | Yes |
| 130 | SAT1 |  |  | 9279 | -0.224 | -0.1186 | Yes |
| 131 | CDKN1A |  |  | 9301 | -0.230 | -0.1000 | Yes |
| 132 | CTSD |  |  | 9320 | -0.246 | -0.0796 | Yes |
| 133 | CD81 |  |  | 9356 | -0.281 | -0.0579 | Yes |
| 134 | MXD4 |  |  | 9373 | -0.310 | -0.0315 | Yes |
| 135 | APP |  |  | 9412 | -0.426 | 0.0030 | Yes |
Table: GSEA details [plain text format]

  

Fig 2: HALLMARK\_P53\_PATHWAY: Random ES distribution      
 Gene set null distribution of ES for **HALLMARK\_P53\_PATHWAY**
